# Shaping Blended Care: Adapting an Instrument to Support Therapists in Using eMental Health

**DOI:** 10.2196/24245

**Published:** 2020-11-13

**Authors:** Hanneke Kip, Jobke Wentzel, Saskia M Kelders

**Affiliations:** 1 Centre for eHealth and Wellbeing Research Department of Psychology, Health & Technology University of Twente Enschede Netherlands; 2 Department of Research Transfore Deventer Netherlands; 3 Research Group IT Innovations in Health Care Windesheim University of Applied Sciences Zwolle Netherlands; 4 Optentia Research Focus Area North-West University Vanderbijlpark South Africa

**Keywords:** eHealth, blended care, implementation science, participatory development, forensic psychiatry, mobile phone

## Abstract

**Background:**

Although eMental health interventions, especially when delivered in a blended way, have great potential to improve the quality and efficiency of mental health care, their use in practice lags behind expectations. The Fit for Blended Care (FfBC) instrument was developed to support therapists and clients in shaping blended care in a way that optimally fits their needs. However, this existing version cannot be directly applied to specific branches of mental health care as it is too broad and generic.

**Objective:**

The goal of this study is to adapt the existing FfBC instrument to fit a specific, complex setting—forensic mental health care—by means of participatory development with therapists.

**Methods:**

The participatory process was divided into 4 phases and was executed by a project team consisting of 1 manager, 3-5 therapists, and 1 researcher. In phase 1, general requirements for the adaptation of the existing instrument were discussed in 2 focus groups with the project team. In phase 2, patient-related factors that influence the use of an existing web-based intervention were elicited through semistructured interviews with all 18 therapists working at an outpatient clinic. In phase 3, multiple focus groups with the project teams were held to create the first version of the adapted FfBC instrument. In phase 4, a digital prototype of the instrument was used with 8 patients, and the experiences of the 4 therapists were discussed in a focus group.

**Results:**

In phase 1, it became clear that the therapists’ main requirement was to develop a much shorter instrument with a few items, in which the content was specifically tailored to the characteristics of forensic psychiatric outpatients. The interviews showed a broad range of patient-related factors, of which 5 were used in the instrument: motivation for blended treatment; writing about thoughts, feelings, and behavior; conscientiousness; psychosocial problems; and social support. In addition, a part of the instrument was focused on the practical necessary preconditions that patients should fill by themselves before the treatment was developed. The use of the web-based prototype of the instrument in treatment resulted in overall positive experiences with the content; however, therapists indicated that the items should be formulated in a more patient-centered way to encourage their involvement in discussing the factors.

**Conclusions:**

The participatory, iterative process of this study resulted in an adapted version of the FfBC instrument that fits the specific forensic context and supports shared decision making. In general, the adaptiveness of the instrument is important: its content and implementation should fit the type of care, the organization, and eHealth intervention. To adapt the instrument to other contexts, the guidelines described in this paper can be followed.

## Introduction

### The Benefits of Blended Care

eMental health interventions are a potentially effective and efficient way to improve the quality of care in a mental health care system that is under pressure due to shortages in staff and money [1-3]. eMental health refers to the use of technology for the treatment or prevention of mental health disorders [4]. Although there are different types of technologies that can be used [4,5], web-based interventions are currently the most predominant form in both research and practice. The content of these types of interventions is based on existing treatment models such as cognitive behavioral therapy or mindfulness, and they offer treatment via multiple modalities such as written text, assignments, and short videos [6]. Studies have shown that these types of interventions can result in clinical outcomes that are comparable with those of standard in-person treatments [7-10]. In addition, they have the potential to increase the efficiency of care by replacing parts of the in-person treatment by web-based treatment [3,11]. Combining this *offline* in-person treatment with *web-based* technologies in mental health care is referred to as blended care [12]. By integrating both approaches, we can have the best of both worlds: offering low-threshold web-based treatment, independent of place and time, which increases the patient’s sense of ownership while maintaining the advantages of a strong therapeutic alliance of in-person treatment [3,11,13]. Despite the benefits of blended care, implementation in practice is very challenging [11,14-16], partly due to the barriers experienced by therapists [17]. Among other things, they often do not think of introducing the possibility of using eMental health interventions to their clients as these are *not in their system* and thus are not on top of their mind [17,18]. Furthermore, especially therapists with little eMental health experience are unsure about the topics that they need to address when introducing or discussing the use of eMental health interventions in treatment with a patient [15,17]. In addition, therapists often decide whether to use eMental health by themselves, based on their own estimation of the patient, instead of considering its applicability together with the patient via shared decision making [18]. If eMental health interventions are used, they are often viewed as a separate addition instead of an equal, fully integrated part of the treatment [19]. Blended care is often delivered in a standardized *one-size-fits-all* way, whereas ideally, the way eHealth is integrated in treatment should be personalized based on characteristics and preferences of individual clients [14,20]. These reasons for the lack of successful implementation of blended care indicate that there is a need to support therapists in shaping their blended treatment in such a way that it can be embedded in treatment to fit the preferences and skills of the patient.

### Fit for Blended Care Instrument

A tool that was designed to support therapists in mixing web-based and offline mental health care is the *Fit for Blended Care* (FfBC) instrument. This instrument aims to support shared decision making in shaping blended treatment in mental health care [12]. To achieve this, it provides topics for therapists and patients to think about and discuss as well as decide on topics related to the needs, characteristics, and skills of a patient regarding blended care. On the basis of a literature review, multiple focus groups and interviews with both therapists and clients as well as a document containing instructions for and the items of the FfBC instrument were created [12]. The instrument consists of 4 main parts, which are briefly described in Table 1. All items of the instrument are provided in the left column of the table in Multimedia Appendix 1. Although the FfBC instrument is considered to be a valuable tool to shape blended care in practice [12,20,21], its current factors seem to be too generic and broad for application in specific domains of mental health care [20]. For example, there are many differences in patient characteristics and treatment goals in the treatment of addiction, anxiety and mood disorders, or delinquent behavior. If the instrument does not optimally fit the characteristics of patients, therapists, and health care, its applicability in practice is low. This implies that there is a need for multiple versions of the FfBC instrument, each adapted to the characteristics of different types of mental health care settings.

**Table 1:** A brief description of the 4 parts of the Fit for Blended Care instrument.

Part of the instrument	Number of items	Examples
Part 1: Practical, necessary prerequisites that need to be met to be able to start blended treatment	A total of 4 items for the patient and 4 items for the therapists to be filled out individually before starting the treatment	Items on clients’ access to a computer; their internet skills; and the presence of acute, severe psychiatric or medical problems that would hinder the use of blended care
Part 2: Possible barriers that might hinder blended treatment	A total of 10 items filled out by therapist and patient together during a treatment session	Items on topics such as a client’s cognitive problems or sensitivity to a psychological crisis
Part 3: Possible facilitators that can facilitate blended treatment	A total of 5 items filled out by the therapist and patient together during a treatment session	Preference for blended care because of practical reasons and a client’s discipline and social support
Part 4: An overview of the previously discussed barriers and facilitators	N/A^a^	An overview of the first 3 parts to prompt therapists and clients to discuss and decide on the composition of blended treatment

^a^N/A: not applicable.

### Blended Care in Forensic Mental Health Care

An adapted version of the FfBC instrument would be especially relevant for the treatment of forensic psychiatric patients. Forensic mental health care is a complex branch of mental health care. The main difference between forensic and regular mental health care is that the main goal of forensic mental health care is to prevent delinquent behavior; therefore, treatment takes place at the intersection between law and psychiatry [22]. Forensic mental health care focuses on the treatment of patients who have committed or are on the verge of committing an aggressive or sexual offense, fully or partly caused by a psychiatric disorder [23]. The use of eMental health in this unique domain appears to be very challenging, which can partly be explained by the characteristics of the forensic psychiatric patients population. Many patients have hardly had any education and have difficulties with reading or writing. Furthermore, forensic psychiatric patients have a broad range of disorders and have committed different types of offenses [24], making the current predominant one-size-fits-all approach toward eMental health interventions not very applicable [25]. In addition, as treatment is often part of a sentence and thus obligatory, many patients are not motivated to be in therapy [24], making it even harder to engage them in eHealth interventions. By integrating eHealth interventions in treatment, the quality of forensic mental health care can be improved, for example, by tailoring eHealth interventions to patient characteristics, by adding persuasive elements that can increase engagement and adherence, or by offering new ways for patients to work on their treatment [25,26]. However, as is the case in mental health care in general, successful implementation of eHealth interventions in existing treatment is considered to be a major barrier [18,26,27].

### Objective

Many branches of mental health care have much to gain from successful blended treatment; however, implementation is a main barrier. To overcome this barrier, the FfBC instrument can be a useful tool. However, to ensure that the FfBC instrument fits the characteristics of a specific form of mental health care, the existing version needs to be adapted. In line with the recommendations on eHealth development, this should be done in close collaboration with end users to ensure that it fits their needs and wishes [28-31]. Consequently, the goal of this study is to adapt the existing FfBC instrument to fit forensic mental health care by means of participatory development with therapists. This will result not only in a new, ready-to-use version of the instrument for forensic mental health care but also in a blueprint for steps that need to be taken to adapt it to other types of (mental) health care.

## Methods

### Setting

This study took place in a Dutch organization that offers forensic mental health care to both inpatients and outpatients. The organization has 2 main outpatient clinics where approximately 85% of all patients are treated. This study took place in one of these outpatient clinics that treats approximately 50% of the organization’s entire patient population. The patient population of this clinic is characterized by a relatively low education level: 46% had only primary and/or secondary education. Furthermore, patients had a broad range of diagnoses, such as personality, attention deficit, sexual, anxiety, depression, schizophrenia, and substance use disorders.

The focus of this project was to adapt the FfBC instrument to an eMental health intervention that was already used by the organization: a web-based intervention platform that contains a collection of over 200 different modules, developed by a Dutch commercial company. The platform is suitable for all types of mental health care and consequently contains modules on, among other things, mindfulness, depression, substance abuse, aggression regulation, relaxation exercises, and social skills. Each module consists of multiple sessions that are provided in a fixed order and can be accessed via a browser or mobile app. These sessions consist of a combination of elements, for example, written information about the topic, a story from a peer (in video or text), written assignments derived from cognitive behavioral therapy, and informative videos. Within the clinic, these modules are used in a blended manner. This means that therapists must first introduce the intervention platform to a patient and select an appropriate module. During usage, the patient is asked to complete assignments in the modules by themselves, on which the therapist must then provide written feedback via the platform in between in-person sessions. Log data analysis has shown that the intervention has been used for over 5 years; however, the uptake in practice is considered disappointing: only 18% of the patients started a module, whereas the goal was to use the intervention with all patients. In addition, among the patients who started, 82% did not finish the module and thus can be characterized as nonadherent. Furthermore, only half of the organization’s therapists used the intervention, of which most used it only several times [18].

### Study Design

Several methods have been used to adapt the existing FfBC instrument to optimally fit the treatment of forensic psychiatric outpatients. The existing version can be found in a paper by Wentzel et al [12], and a summarized version is provided in Table 1. To create an adapted version, an agile approach was applied, in which several subproducts were created, regularly evaluated with therapists, and adapted accordingly [32]. These formative evaluation cycles are in line with current recommendations on eHealth development and support developers in ensuring that the final product fits the needs and characteristics of the end users and their contexts [33]. The phases of this study and the accompanying methods used are presented in Table 2.

**Table 2:** An overview of the methods used to adapt the Fit for Blended Care instrument for forensic mental health care.

Research method		Main research goal	
**Phase 1: Requirements for adaptations**			
	A total of 2 focus groups with 3 therapists and 1 manager		Identifying the preferences and ideas of the therapists, managers, and researchers to determine the general layout and structure of the to-be-adapted FfBC^a^ instrument
**Phase 2: Identifying factors**			
	Semistructured interviews with all 18 therapists of 1 outpatient clinic		Identifying specific forensic psychiatric patients–related factors that influence the use of the eMental health intervention
**Phase 3: Content generation**			
	Focus group with 3 therapists, 1 manager, and 2 researchers		Formulating the items that should be integrated in the to-be-adapted version of the instrument, based on previously identified factors
	Focus group with 6 therapists, 1 manager, and 2 researchers		Formulating tips and recommendations for therapists on how to deal with different types of patient-related factors
	Prototyping		Developing a functioning, interactive prototype of the adapted version of the FfBC instrument
**Phase 4: Testing**			
	Pilot study with 5 therapists		Gaining insight into the experiences of therapists and practical feasibility of using the instrument
	Focus group with 5 therapists		Identifying points of improvement for the adapted version of the FfBC instrument
	Prototyping		Developing an improved version of the FfBC instrument based on the points of improvement of previous phases

^a^FfBC: Fit for Blended Care.

Throughout the entire process, a project team was actively involved. This team consisted of the researcher who led the focus groups (HK), a minimum of 2 and a maximum of 5 therapists, and the team manager. Therapists were included by the team manager based on their motivation to improve the use of eMental health. To ensure different perspectives, not all participating therapists were very positive about the intervention. All members had at least three years of experience working in mental health care, and all had used the eMental health intervention at least once. The composition of the project team changed throughout the process for various reasons: 1 member was replaced by another due to personal circumstances, and later in the process, 3 new members were added to expand the expertise and experience of the project team. In addition, not all members could join all focus group meetings due to conflicting appointments.

### Materials and Procedure

#### Phase 1: Requirements for Adaptations

As can be seen in Table 2, the goal of the first phase was to map the requirements, that is, the needs and wishes of the involved therapists regarding adaptations to the existing version of the FfBC instrument (Table 1).

##### Participants

In the first phase of the process, two 1-hour focus groups were held with 1 manager and 3 therapists—of which 1 was a social worker and 2 were psychologists; all were members of the project team.

##### Data Collection

In the first focus group, the participants studied the existing FfBC instrument and discussed its potential usefulness to determine whether adapting it would be of added value for the organization. After agreeing on its usefulness, the second focus group focused on the therapists’ needs and wishes regarding an adapted version of the instrument via a brainstorming session about required adaptations. The main discussion points centered on the content of the items, the length of the instrument, the way of filling it out, and the way in which the questions were asked.

##### Analysis and Product

On the basis of the notes that were taken by the researcher (HK), a document was created with the stakeholders’ requirements regarding the adapted version of the FfBC, which was checked and verified by the participating therapists.

#### Phase 2: Identification of Factors

To create the content of the adapted version of the FfBC instrument that was specifically tailored to the use of the web-based modules in forensic mental health care, semistructured interviews with therapists were conducted to gain insight into the patient-related factors that, according to the therapists, are related to the use of the web-based modules.

##### Participants

To avoid self-selection bias, all 18 therapists working at the forensic outpatient clinic were included in the interview study. All therapists were expected to use the eMental health intervention when offering therapy. The included therapists had different occupations: 8 psychologists, 6 social workers, 2 system therapists, 1 trauma therapist, and 1 forensic nurse were interviewed. At the time of interviewing, they had been working in forensic care for an average of 13.18 years (SD 8.68), with a range of 8 months to 29 years.

##### Data Collection

After the interview’s goal and content were discussed, informed consent was obtained. The entire interview consisted of 6 main categories with open-ended questions: (1) sociodemographic questions, (2) experiences with the introduction of the intervention, (3) the way in which the intervention was used with patients and/or reasons for not using the intervention, (4) the potential added value of the intervention, (5) the ideal situation regarding implementation in practice, and (6) barriers related to the use of the intervention. Consequently, patient-related factors were discussed throughout the interview.

##### Analysis and Product

To identify patient-related factors, an inductive, bottom-up approach was applied to analyze the transcripts. For this study, 195 fragments on patient-related factors that are related to the use of the eMental health intervention were identified. Next, an initial coding scheme was created based on these fragments using the method of constant comparison [34]. Overall, 2 researchers used the initial coding scheme to code 20% of the fragments, resulting in a joint probability agreement of 89%. No further adaptations to the underlying structure of the code scheme were required. Owing to the high interrater reliability, 1 researcher coded the remaining fragments and discussed them with the other researcher in case of doubt.

#### Phase 3: Content Generation

As shown in Table 3, the goal of the third phase was to combine the requirements of phase 1 and patient-related factors identified in phase 2 into an adapted version of the FfBC instrument that fit the needs and wishes of the therapists and the characteristics of forensic outpatient care. This was achieved in 2 stages, of which the first stage focused on the creation of items for the instrument and the second stage on the tips and guidelines that the instrument should offer. This resulted in the creation of a functional prototype of the FfBC instrument.

**Table 3:** Main codes and the number of interviews in which they were identified (Nint=18) and the total number of times that a code was identified (Ntot=195).

Main code	Interviews in which the main codes were identified, n	Total number of times the code was identified, n
Treatment motivation	14	40
Conscientiousness	14	27
Literacy levels	14	22
Perceived benefits	14	22
Psychosocial situation	13	28
Technological skills	12	17
Availability of technological resources	11	18
Reflective skills	11	21

##### Participants (Focus Groups Round 1)

A total of 2 researchers, 5 therapists, and 1 manager participated in the first focus group.

##### Data Collection (Focus Groups Round 1)

A 1-hour focus group took place to discuss which patient-related factors identified in phase 2 should be included in the adapted FfBC instrument. One researcher led the focus group by explaining the previously identified factors and asking the participants whether these factors would be suitable for inclusion in the instrument.

##### Analysis and Product (Focus Groups Round 1)

On the basis of the discussion, 2 researchers created a table with (1) the factors from the original instrument, (2) comparable or similar factors from the previously conducted interviews, (3) a suggested adaptation for the adapted version of the FfBC instrument, and (4) substantiation and explanation for the adaptation. Furthermore, for each factor, 3 *multiple-choice* options to indicate the extent to which a factor was estimated to be present within a patient were added. A document with the factors, a brief explanation, and the 3 options were discussed with 5 therapists and 1 manager in a new focus group and adapted accordingly.

##### Participants (Focus Groups Round 2)

In the second focus group, the same 5 therapists and the manager participated, and 1 researcher was present.

##### Data Collection (Focus Groups Round 2)

A focus group was conducted with the project team (all 5 therapists, a researcher, and a manager) and 1 additional researcher who was actively involved in developing the existing version of the FfBC instrument. In this focus group, concrete tips and guidelines on how to deal with specific patient-related factors were generated. All therapists participating in the focus group had experience using the intervention and were asked to use their own experiences to formulate the tips and recommendations. The researcher and manager also actively participated in the brainstorming session. Each factor was discussed separately, and general tips and guidelines were discussed as well.

##### Analysis and Product (Focus Groups Round 2)

The researcher kept extensive notes. On the basis of these notes, a document with tips per patient-related factor was created. This document was validated by the participants of the focus group, and several minor adaptations were made accordingly. This resulted in 1 document with all tips and guidelines that had to be integrated into the adapted FfBC instrument. On the basis of the previously identified items and the tips that were generated in phase 3, a working prototype of the instrument was created in Qualtrics (SAP SE), a web-based survey system. In this prototype, therapists were able to select 1 answering option per patient-related factor, resulting in tailored advice for each factor.

#### Phase 4: Testing

The goal of the fourth phase was to gain insight into the experiences and identify points for the improvement of the functioning prototype of the FfBC instrument by testing it in practice.

##### Participants

In the pilot study, 5 therapists were asked to use the prototype of the FfBC instrument with 3 patients per participating therapist, resulting in an intended number of 15 patients. A total of 4 therapists participated in the focus group.

##### Data Collection

In total, the instrument was used with 8 patients. In the focus group, 4 therapists were asked about their experiences with the content, usability, and integration in treatment. To ensure that all relevant topics were discussed, a semistructured approach was used in which the following topics were discussed: the way in which the instrument was used, opinion about the instrument, and recommendations for improvement.

##### Analysis and Product

On the basis of the outcomes of the focus group, changes were made to the initial prototype to ensure an optimal fit with the needs and wishes of therapists, which was again evaluated by the therapists. This version of the instrument will be further developed and implemented in clinical practice.

## Results

In this section, the results are discussed for each of the 3 phases (Table 2) and their accompanying research methods.

### Phase 1: Requirements for Adaptations

The first focus group showed that therapists saw the potential of the FfBC instrument in addressing the current implementation problems. On the one hand, it was seen as a way to offer concrete and relevant topics to discuss to identify the most optimal way to shape blended care. On the other hand, if implemented well, the instrument could be seen as a *reminder* that could help therapists in remembering to bring up the use of technology, as therapists often forgot to introduce the possibility of blended care or decided for themselves that a patient would not benefit from eMental health. However, participants indicated that the instrument needed to be adapted to better fit the forensic context and to account for several practical limitations.

The second focus group resulted in the following broad requirements for the new version of the FfBC instrument:

- The adapted version should be shorter and contain fewer texts and fewer items. Therapists found that the existing instrument contained too many items and thus would be too time consuming.
- Each item of the adapted version should be accompanied by 3 to 4 multiple-choice options. Therapists indicated that open-ended questions would require too much time.
- Each multiple-choice option should be accompanied by tailored advice and tips and tricks specific for that option. Therapists indicated that these tips and tricks could support them in initiating and continuing the use of the modules.
- The items of the existing version need to be specified to fit the treatment of forensic psychiatric outpatients. Therapists found the items in the existing version too broad and generic for use in forensic mental health care; therefore, the adapted version of the instrument should be based on patient-related factors that specifically influence the use of eHealth interventions in forensic mental health care.
- There should be a web-based version of the instrument. Therapists believed that a web-based version would be easier to fill than a paper-based version.
- Patients have to answer several questions about the necessary preconditions for using eMental health in advance by themselves. Therapists stated that this could avoid them from discussing these practicalities in treatment, which would demand valuable time. This means that therapists wanted to keep the existing distinction between the first part and the additional items of the instrument [12], where the first part should be filled out by the patient and the second part should be discussed by the therapist and patient together.

### Phase 2: Identification of Factors

The patient-related factors that, according to therapists, influence the use of the eMental health intervention that arose from the interview study are presented in Table 3.

#### Treatment Motivation

Motivation refers to the extent to which a patient is enthusiastic or open toward working with the eMental health intervention in treatment. Although some patients were described as motivated for blended treatment, therapists indicated that a large proportion of the patients were not eager to work with the eMental health intervention. A lack of motivation was not only observed at the beginning of the blended treatment but also when the patients were using the intervention. This lack of motivation is illustrated by the following quote:

But I think it will be very difficult for a patient who already is not very motivated, to also encourage him to log in again, and to read things again, because there’s a lot of text sometimes. And to work on assignments. It would be better to lower the threshold a bit at first.

#### Conscientiousness

Conscientiousness refers to the extent to which a patient adheres to agreements regarding the independent use of the intervention outside of treatment, which was described by 1 participant as follows:

But you have to actually do it, you really have to get into it. And even though they can practically do it, they still have to put their mind to it. Plan a moment for it, do things, take steps. And a lot of patients don’t get to that point.

PP 3

Therapists indicated that it often required a lot of their time and effort to ensure that patients performed the activities that they agreed on, such as working on and completing assignments. A comparison was made with doing homework, with which a large share of the patients, who often received little education, had difficulties.

#### Literacy Levels

Literacy levels refer to the patient’s ability to write, read, and understand treatment-related information in the intervention. One therapist described this problem in the following way:

But you do meet people who cannot even write. I don’t want to call them illiterate, but they are very ashamed of a lot of linguistic errors and things like that. That’s a barrier with which you’d have to help them first, so that it isn’t about the sentence construction or errors, but what’s going on in their head. Just try to write that down in your own words. And people often find that difficult.

PP 10

In addition, therapists indicated that patients had difficulties not only with writing but also with reading, as the intervention contained several words that were perceived as difficult.

#### Perceived Benefits

Perceived benefits refer to the extent to which a patient experiences or expects to experience a positive influence on his or her treatment because of the use of the technology. Therapists indicated that if patients did not directly see how a module fits their problems or could be of added value for them, the chances of them using the module were lower. A therapist said the following about this:

There can be a lot of reasons for that. It might be that some have heard about it from others, that it’s helpful. Or that some modules fit well. And also what I’ve said before, that it fits the needs of the patient. So if you offer a sleeping module for someone with difficulties with sleeping, there’s a greater chance that he will continue.

#### Psychosocial Situation

Psychosocial situations refer to difficult circumstances or events in a patient’s personal life and/or mental state that influence the use of technology for treatment. This can refer to patients who are in a crisis such as a psychosis or severe depression and to those with problems related to their daily life, such as fights with neighbors or loved ones, no current place to stay, or money problems. This is further illustrated by the following quote:

For two patients it wasn’t possible to complete the assignments. And one of them is someone of whom I think, there’s just too much going on. That person has lost his job, the emotions are all over the place, and that makes it more difficult to work on a session, even though it might be beneficial.

Overall, therapists indicated that it is important for patients to have a relatively steady life when using the intervention, because they otherwise have no *mental space* to work on the module.

#### Technological Skills

This code refers to the level of skills required for successful use of information and communication technologies such as computers or smartphones. Therapists indicated that several patients, especially older ones, have difficulties with using technologies. These difficulties could be with either using the actual technology, such as a computer, or navigating through the intervention itself. One therapist said the following:

I can definitely imagine that with young people, who already sit behind the computer a lot, it might fit a bit better. I can really imagine that.

PP 3

#### Availability of Technological Resources

This code refers to the patient’s access to a technological device, an appropriate working area, and a good internet connection that is necessary to use the technology. The importance of a suitable work space is described in the following quote:

I think that in their own environment, where they like doing it. They have to be able to do it privately, not that there is someone around the entire time. So privacy is important for them, I think. We can’t facilitate that; they have to arrange that themselves. Or we’d have to offer them a place to work here, so they can sit behind a computer here.

PP 7

#### Reflective Skills

Reflective skills refer to the patient’s ability to independently reflect on and write about emotions, cognitions, and behaviors in the technology. Often, patients are not used to talking about their problems, and writing individually about these situations is often even more difficult. Therapists also indicated that reflecting individually could also lead to intense emotions and adverse consequences because of a patient’s inability to independently deal with them, as explained in the following quote:

And also that it elicits too much emotions that they cannot directly deal with by talking to someone. Basically, you’d have to inhibit the direct gratification of your own needs. Yes, they can chat, but they do not receive an answer immediately. And some patients keep on thinking about it, running it through their head, because they do not get support directly.

PP 16

### Phase 3: Content Generation

In the first focus group of this phase, 3 therapists, 2 researchers, and a manager decided on items that should be integrated in the instrument based on the factors identified in phase 2. In line with the requirements identified in phase 1, the number of factors that arose from the interviews needed to be reduced. To create an overview of relevant items, a table was created by 2 researchers, in which the factors of the original instrument were combined with the suggested change to the item and a substantiation of the change by means of the results of the previous phases. In Table 4, a part of this table is provided to illustrate this process. The complete table can be found in Multimedia Appendix 1.

**Table 4:** Examples of the table used to create items of the adapted version based on the original version of the Fit for Blended Care instrument.

Item from original version	Item for adapted version	Rationale behind change
10. *Motivation and trust* (discuss and answer): Do you (client) trust that a blended treatment can help you with your complaints? Are you (client) motivated to do a blended treatment?	*Motivation forweb-basedtreatment:* to what extent is the patient motivated to work on the eMental health intervention in his or her treatment	Motivation is an important issue in forensic mental health care according to the interviews Lack of trust in effectiveness was not an important topic in the interviews; therefore, remove it for conciseness Rephrase item because the instrument has to be filled out by a therapist (after discussing with the patient)
15. *Working alliance*: Is there a good working alliance or do you (therapist and client) expect that a good working alliance will be developed? Note: Here it is important that you (the client) recognize your own contribution to the therapy and are aware of what is expected of you.	N/A^a^	Remove to make the instrument more concise Person administering this instrument might be someone other than the therapist (eg, the *intaker*) Hard to assess in the first meetings, especially in forensic patients who are obliged to attend treatment; they might have a different attitude than later in the treatment process Not an important topic in interviews

^a^N/A: not applicable.

A total of 2 researchers (HK and JW) combined the findings from the interviews with the factors from the existing instrument, which resulted in 5 items. The researchers also created 3 *multiple-choice* answering options from which a therapist had to choose the most fitting option. These 5 items and multiple-choice options were combined into a document with the first version of the adapted FfBC. This document was checked by 3 therapists from the project team, and slight changes in phrasing were made accordingly. The way in which the instrument should be administered was discussed in the focus group. It was decided that the first part, focusing on the practical preconditions, should be filled out by a patient individually, ideally before beginning the treatment. The second part should be filled out by the therapist based on a discussion of all 5 factors with the patient, either at the beginning or during treatment. A summarized version of the instrument is provided in Textbox 1; the text of the entire adapted version of the instrument can be found in Multimedia Appendix 2.

**The main topics and summary of the content and questions of the adapted Fit for Blended Care instrument.**

Content of part 1: Necessary preconditions

- Reading and writing
  Are you able to read and write short texts?
- Workstation and devices
  Do you have access to a device (computer, laptop, smartphone, or tablet), does it have a well-functioning internet connection, and is there a place where you can work on web-based treatment in a calm and familiar manner?
- Internet skills
  Are you able to send emails, watch videos on the web, use the internet to read short texts, use social media, and use the internet to send messages to others?

Content of part 2: Patient-related factors that can influence blended care

- Motivation for blended treatment
  To what extent is a patient motivated to work with the eMental health intervention in his or her treatment?
- Writing about thoughts, feelings, and behavior
  To what extent is a patient able to independently write and reflect on his or her thoughts, feelings, and behavior?
- Conscientiousness/working with discipline
  To what extent is a patient capable of sticking to appointments on blended care for matters such as forgetfulness, concentration, or planning skills?
- Psychosocial problems
  To what extent are there problems in the patient’s private life and/or severe psychiatric problems that can have a negative impact on using the eMental health intervention?
- Social support
  To what extent does a patient have a social network (partner, parents, and friends) that is able to support him or her in using the eMental health intervention?

In the second focus group, the therapists, researchers, and a manager formulated tips and recommendations for therapists on how to deal with different types of patient-related factors, resulting in a document with tips for all 5 items of the second part of the instrument. Each multiple-choice answering option was accompanied by tailored advice specific to that option. On the basis of the outcomes of the focus group, a researcher (HK) created a document with the recommendations, which was emailed to the researcher, therapists, and manager. On the basis of their input, several minor changes to phrasing were made. In Textbox 2, one tip or recommendation per item is provided for illustration purposes. To prevent therapists from using the instrument as a reason for not using eMental health interventions, the members of the focus group decided that the tips should never suggest not to use eMental health. Instead, the tips should encourage therapists to think outside the box to involve difficult-to-engage patients in blended care or to delay the use of eMental health to a later point.

**Examples of advice or recommendations provided per item of the instrument.**

General advice

- Before you start with a module, it is important to discuss with the patient what the added value of the module should be. Make sure you set clear goals that you both agree on. On the basis of that, you can regularly evaluate how the blended treatment is going.

Motivation for blended treatment

- When a patient is not motivated at all, it is important to figure out why this is the case by means of an open discussion, instead of just accepting it. It might be that a patient foresees obstacles that are actually easy to overcome.

Writing about thoughts, feelings, and behavior

- You can take away a patient’s fear for writing by clearly stating that you do not expect flawless spelling or elegant phrasing but that the goal is to think about thoughts, feelings, and behaviors. You might suggest the patient to use very short sentences or terms.

Conscientiousness/working with discipline

- If the patient did not hand in an assignment although this was agreed on, you can send a reminder to ask the patient why he or she has not completed the assignment yet and if he or she is able to still finish it.

Psychosocial problems

- If a patient is experiencing a crisis such as current psychosis or suicidality, it is often not recommended to directly start with an eMental health intervention because the crisis has to be dealt with first. However, it is possible to start the intervention at a later point. It is advised to regularly evaluate with the patient to determine if blended treatment can be initiated after a while.

Social support

- If a patient indicates that one or more loved ones can actively support him or her, you can look for possibilities to actively involve those in blended treatment. A loved one might support the patient in working on assignments.

The final activity of the third phase was the development of a working prototype of the instrument to enable therapists to test it with several patients. A digital prototype was created based on the needs and wishes of the therapists that were identified in phase 1. The prototype was made in Qualtrics, as this software provides tailored advice per chosen answering option.

### Phase 4: Testing

The goal of the fourth and final phase was to identify experiences with and accompanying points of improvement of the previously developed prototype. After 2 months, 5 therapists used the instrument with a total of 8 patients. This was about half of the expected 15 patients. The most important experiences and conclusions of the final focus group in which the prototype was evaluated are as follows:

- Therapists indicated that the 5 factors were useful to discuss and that the current content sufficed: no factors should be removed or added.
- The first part of the FfBC instrument was considered as useful, but therapists indicated that it was difficult to remember to ask the patients to fill it out beforehand. It was considered important to integrate the first part in existing structures, for example, in a web-based
  welcoming module
  .
- The second part of the FfBC instrument was used several times but not as often as expected, as therapists were asked to try the second part with at least three patients. The main explanation for this was that they did not remember to administer the instrument during their treatment routines. They indicated the importance of reminders to support them in remembering the use of the FfBC instrument in treatment. Other reasons for the lower usage were not provided; it was mostly attributed to not remembering to use the instrument, and therapists expressed the intention to use it more.
- Therapists indicated that the instrument can be of added value during multiple points in treatment. For example, it can be used at the beginning of the treatment to get an idea of the type of patient and to plan blended care; however, it can also be used throughout the course of the treatment, for example, if a patient stops using a module or if the use of the module is not going as expected.
- The prototype was designed in such a way that therapists had to fill out the instrument individually, after discussing the factor with the patient. However, therapists indicated that, in practice, they preferred to fill the instrument together with the patient and expressed a need for a patient-centered version, including easier phrasing.
- In addition to a web-based version, several therapists expressed the need for a paper-based version that they could fill out together with the patient when, for example, no laptop was available in case of home visits or if they preferred not to sit behind their computer with the patient.
- To use the instrument, therapists had to use the Qualtrics prototype, which was considered inconvenient as they often could not retrieve the link, which was e-mailed to them. They indicated that it would be easier to integrate the instrument in one of the existing systems they used, among which were the platform of the eMental health intervention and the electronic patient file. Consequently, the importance of integrating the instrument in these systems was emphasized to prevent the use of the instrument as an additional time-consuming activity.

In the focus group, the project team decided that the first part of the FfBC instrument should be integrated in a to-be-developed web-based *welcoming module*, which is expected to be followed by all patients that start the treatment. The questions of the second part of the FfBC instrument have to be integrated into an existing system that has all existing questionnaires that are used in treatment to ensure that the FfBC is used in the same way as other questionnaires that are used in forensic mental health care and to ensure that they do not require any additional work. Furthermore, based on the outcomes of this focus group, a second patient-centered version of the FfBC instrument was developed; the content and a screenshot of the instrument are provided in Multimedia Appendix 3. In this additional version, the phrasing of the 5 factors is targeted at patients, that is, shorter and simpler sentences without jargon. Participants of the focus group indicated that these items can be printed on paper, for example, in the form of a poster or as 5 separate cards that can be placed on the desks of therapists, to ensure that the items are always visible, which was expected to serve as an additional reminder. Together, the patient and therapist can discuss these items during a therapy session to determine which answering option best fits the patient, instead of the therapist deciding individually on which option fits best afterward.

## Discussion

### Principal Findings

This study described the development of an adapted version of the existing FfBC instrument to optimally fit forensic mental health care. In phase 1, it became clear that therapists wanted a shorter, easier-to-use version of the instrument, ideally on the web, containing factors that were more specific for forensic mental health care. In phase 2, a broad range of patient-related factors were identified in a systematic interview with all therapists working at the outpatient clinic. In phase 3, these factors were translated into a functioning prototype of the instrument, using the broad requirements from phase 1. The instrument consisted of 2 parts: one to be filled out by the patient individually, targeting practical necessary preconditions, and one with 5 items that should be discussed by the patient and therapist to shape blended treatment. These factors were motivation for blended treatment; writing about thoughts, feelings, and behavior; conscientiousness/working with discipline; psychosocial problems; and social support. In phase 4, the prototype was used in practice. The adapted version of the instrument was seen as useful and promising but was not used as often as expected in the pilot study. Therapists indicated that the main reason for this was a lack of integration in existing systems and procedures, showing that a fit between the instrument and their current practices was deemed essential for its success and added value for clinical practice. On the basis of the outcomes of this final phase, a second, more patient-centered version was developed, with items that are phrased in a shorter and simpler manner.

### Adapting the Instrument

Although in this study an adapted version for the use of a web-based intervention platform in forensic mental health care was created, the FfBC instrument can be adapted to fit many different types of mental health care and even for other types of health care where eHealth interventions are used, such as physiotherapy [20] or by general practitioners. This study showed that to prevent an overload of factors resulting in an impractical and time-consuming instrument, only the most important ones should be included. Patient-related factors that are most important might differ per branch of (mental) health care. For example, the interview study and focus groups showed that conscientiousness is seen as a very important topic for forensic psychiatric patients: therapists stated that they often have difficulty working independently on modules and doing their *homework* [6,18]. However, this issue might be less relevant in other domains of mental health care. To illustrate, it is known that highly educated women are most adherent to eHealth interventions [35,36], and although these types of patients are underrepresented in forensic mental health care, they are more prevalent in the treatment of, for example, anxiety or mood disorders [37]. This might imply that conscientiousness is a less relevant factor in that domain. Consequently, the version of the instrument that was developed in this study cannot be copy-pasted to be used in other settings.

To adapt the instrument to ensure that it fits a specific form of health care, the approach used in this study can be used as a guideline. Each new project should start with the generation of general requirements regarding adaptation, either using the original elaborate version of the FfBC Instrument (Multimedia Appendix 1) or the version developed in this study (Multimedia Appendices 2 and 3). In phase 2, we conducted a semistructured interview to identify the factors. Other projects can apply the same approach; however, as it is quite time consuming to conduct an entire interview study, the factors that were identified in this or other studies on eHealth usage for a specific setting might be used, as long as they are validated with therapists and possibly patients. In phase 3, the actual instrument was developed. The findings of this study can be used as the foundation. For example, other instruments can also use the distinction between part 1, which focuses on practical necessary preconditions and must be filled out by patients themselves, and part 2, which contains approximately 5 items that must be discussed in treatment. However, although the existing and adapted version of the instrument can be used, changes should always be discussed with therapists to ensure a participatory approach. Finally, before implementing the instrument in practice, it has to be pilot tested and changes should be made accordingly, as was done in phase 4 of this study. As became clear in this study, including many patients in a pilot test with a prototype that is not yet integrated in existing systems can be challenging from a practical point of view. However, in usability testing, the general guideline is that 5 to 7 tests are often enough to identify most flaws of the prototype [38]. Possibly, this reasoning can be extended to the pilot test, which might mean that testing the instrument with this number of patients might suffice to identify the most important points of improvement. However, future research should show whether this is actually the case.

In general, this study has shown that developing an adapted version of the FfBC instrument requires multiple phases that are connected by continuous formative evaluation cycles with active end-user involvement. The main challenge for the adaptation of other versions will be to identify an approach that is thorough yet not too time consuming. The guidelines and content that were generated in this study can support other researchers in setting up an efficient yet thorough development process.

### Shared Decision Making

An important finding that gradually became clearer throughout the process of adapting the FfBC instrument was the importance of shared decision making in shaping blended care. In current clinical practice, the decision on whether and how to use eMental health interventions is often made in a top-down manner, with the therapist deciding the intervention that will be used, the frequency of usage, and the mode of communication about it [17]. However, therapists participating in this study clearly expressed the need for an instrument that facilitated shared decision making as much as possible. Although the initial prototype encouraged therapists to discuss the factors elaborately with patients, the therapists had to decide on the most suitable option individually, and the text of the instrument was focused on the therapist. The pilot test showed the need for an additional version to be filled out together with the patient during treatment. In this project, cards with these patient-centered items were developed; however, there are other possibilities to further support shared decision making in shaping blended care, such as digital versions using tablets or mobile phones or gamified versions, which can be developed in further research. Using the FfBC instrument is an excellent way to prevent top-down processes and fits within the models of shared decision making such as that of Elwin et al [39]. Consequently, in line with current movements such as positive health, the use of the FfBC instrument results in a more prominent role of patients in their own health and health care, which can increase their sense of ownership and self-management [40].

### Future Research

This study mostly focused on the development and formative evaluation of a therapist- and client-centered version of the FfBC instrument. Although this instrument is well-substantiated and can be used in clinical practice, more research is required. First, the instrument needs to be used in clinical practice by more therapists and in more organizations to further optimize it. In line with this, it is important to note that this version of the instrument should not be seen as fixed; it should constantly be adapted based on experiences, new insights, and changes in treatment or context. Second, a necessary precondition to further optimize the instrument is that it is actually used in practice. The results of the pilot study showed that, even though therapists saw the items as valuable, it was not used as much as expected beforehand. This touches upon a larger problem related to the implementation of new innovations in clinical practice [17,41]. On the basis of the outcomes of this study, the main reason for this seems to be that the therapists simply forget about using this new instrument during their daily routines. This conclusion is in line with the work on the implementation of eHealth: although health care professionals see the added value of an intervention, they often do not use it because it is *not in their system* and does not seamlessly fit their regular activities [18,42]. However, as there might be a broad range of other reasons for possible low acceptance of the instrument, such as a negative attitude toward using eHealth in general or a lack of skills to fill out the instrument together with the patient [17,43], future research should use the instrument in a larger sample of therapists and investigate the reasons for nonacceptance.

Third, although the main goal of the instrument is to shape blended care in a fitting way, a secondary goal is to help the therapist remember to introduce blended care, as this is often overlooked in day-to-day practice [5]. By using the instrument with all patients, the uptake of eMental health in practice might improve. Further research using log data analyses can study whether the use of this instrument actually results in increased usage and whether different scores on the 5 items can be related to different ways in which a module is used. In addition, it is expected that a better fit between the patient’s needs and a blended treatment will result in better adherence to and effectiveness of the intervention, as personalized interventions can result in improved outcomes [44,45]. However, not much is known about this topic within the domain of blended care; therefore, research that aims to determine whether increased use of the instrument indeed results in higher usage of and engagement with eHealth interventions is recommended.

The instrument has the potential to not only benefit clinical practice but also add value for research on eMental health interventions. In line with the previous recommendation, items of the FfBC instrument might serve as predictors for the effectiveness of eMental health interventions. It appears to be difficult to predict whether and why users are nonadherent to an intervention and whether it is effective for an individual [46,47]. Often, sociodemographic factors are identified as predictors [47]; however, these factors do not provide much information that is useful in clinical practice as they are often fixed, for example, we cannot change someone’s age to increase the effectiveness of the intervention. However, the items of the FfBC instrument might be potential moderators for effectiveness. For example, if the level of motivation appears to be an important predictor of effectiveness, therapists might be encouraged to increase a patient’s motivation for blended care, which might increase the chances of an intervention being effective. An accompanying advantage is that the outcomes of FfBC and log data from clinical practice can be used to identify predictors. These results will have more ecological validity as most research on the predictors of effectiveness is conducted with data from randomized controlled trials, which take place in controlled settings as opposed to data from eHealth use in the *real world*. However, to achieve this, the instrument should be adapted for use as a research tool instead of a clinical tool. Among other things, the items should be accompanied by consistent and validated scoring options, and a study on its reliability and validity as a research tool is needed.

### Strengths and Limitations

The main strength of this study is its iterative nature with multiple formative evaluation cycles. Applying such a bottom-up approach where products are created based on collected data and evaluated with end users results in a final product that is ecologically valid and closely fits the requirements from practice [32,48]. Throughout this process, the main focus of data collection was on therapists as they were the main target group of the instrument. However, the patients were not actively involved. This might have resulted in a bias, overlooking factors that were important for the use of eMental health according to patients. Nevertheless, as most involved therapists had much experience with using eMental health with patients and had discussed reasons for nonusage with their patients, the chance of missing important factors is considered fairly low. In addition, comparable factors have been identified in other studies in which patients and other stakeholders are involved [6,49]. However, it is recommended to actively involve the patient perspective in following research to verify whether the identified items are in line with their experiences as well. Furthermore, data collection took place at one outpatient clinic of one organization. Although this was a deliberate decision to ensure that the adapted version of the instrument seamlessly fitted this organization, the specific focus raises questions about the generalizability of the results. Although the involved therapists had much experience in forensic mental health care, it is still advised to pilot test the instrument and its implementation in other forensic organizations to ensure that it also fits their needs and way of delivering blended care. Finally, another limitation regarding generalizability is related to the country in which this study took place. Although many similarities exist between Dutch mental health care and that of other countries, there are also many differences. This implies that this version of the instrument cannot be copy-pasted into the forensic mental health care of other countries. Therefore, we stress that the instrument should always be adapted to fit specific settings, and this applies to health care in other countries as well. The guidelines developed for adapting the instrument can be used for this purpose.

### Conclusions

This study showed that the iterative, participatory development of an FfBC instrument resulted in an adapted version that fits the context by incorporating the needs and wishes of therapists and patient-related factors that are relevant for the use of web-based interventions in forensic mental health care. This instrument can further support shared decision making in blended care, as this is an important yet often overlooked topic. The instrument’s adaptability is important: its content, design, and implementation in existing care should fit the specific type of health care, organization, and eHealth intervention for which it is used; it is not a one-size-fits-all tool. To adapt this instrument to other contexts, the guidelines described in this paper can be used. By using such approaches to better integrate in-person care and eHealth interventions, we can combine the best of both worlds and increase the quality of care.

## Appendix

Multimedia Appendix 1 The table used to create items of the adapted version based on the original version of the Fit for Blended Care instrument, interviews and focus groups.

Multimedia Appendix 2 Fit for Blended Care instrument – Therapist-centered version.

Multimedia Appendix 3 Fit for Blended Care instrument – Patient-centered version.

## Abbreviations

FfBC: Fit for Blended Care
