# Supplementary material for: Shaping Blended Care: Adapting an Instrument to Support Therapists in Using eMental Health
Source: JMIR Ment Health. 2020 Nov 13;7(11):e24245. doi: 10.2196/24245 (PMC7695535; doi:10.2196/24245)
Supplement: Multimedia Appendix 1 [file mental_v7i11e24245_app1.docx]

# Multimedia Appendix 1

## The table used to create items of the adapted version based on the original version of the Fit for Blended Care instrument, interviews and focus groups.

| Item from original version  (translated from Dutch to English by authors of this paper) | Item for adapted version | Rationale behind change |
| --- | --- | --- |
| Part 1a: Practical and necessary prerequisites that need to be met to be able to start blended treatment (to be answered by client) |  | It is possible to ask these questions in a to-be-developed ‘welcoming module’ in the eMental health platform. If patients don’t use the welcoming module, they should complete the first part during a treatment session (for example the intake). |
| 1. Access to a computer  Do you have access to a computer?  *Clarification:* Also select yes when you don’t have a computer, but if you can use the computer of someone you know. | **Place to work on online treatment**  Do you have access to a device (computer, laptop, smartphone or tablet) on which you are able and willing to work on online treatment?   - Yes, I do have access to a suitable device - No, I don’t have access to a suitable device - No, but I will have access to a suitable device within approximately one month | - Focus group: patients can also work on eMental health interventions on a smartphone or tablet, so we should consider other devices than solely a computer. - An option should be added for patients who don’t have a computer at this point due to circumstances (e.g. just being released from prison, temporary financial problems or homelessness), but will have access to one soon. - Is the term ‘online treatment’ suitable, does it fit with the jargon of the therapists? |
| 2. Internet access  Do you have access to the internet? | **Place to work on online treatment**  Do you have access to a good, stable internet connection?   - Yes, I have access to good internet - No, I don’t have access to good internet - No, but I will have access to good internet within approximately one month | - According to the focus group this item should be made a bit more specific. - An option should be added for patients who don’t have internet access yet, but will have this soon. - The phrasing should be as clear as possible: not just access, but good access for e.g. watching videos. |
| 3. Place to work  Do you have a quiet place where you can work on your treatment in a calm and safe way? | Do you have access to a place where you can work in a calm and pleasant way on your online treatment?   - Yes, I do have access to a suitable place - No, I don’t have access to a suitable place - No, but I will have access to a suitable place within approximately a month | - ‘Safe’ might not be the best term for this patient population (might have another meaning). - An option should be included for patients that will soon have a suitable place to work. |
| 4. Internet skills  React to the following statements (yes/no/don’t know).   - I know how to open saved files. - I know how to save a picture from the internet. - I know how to use keyboard shortcuts (e.g. CTRL-c for copying). - I know how to open a new window in my browser. - I know how I can add a website to the favourites. | **Reading and writing**  In order to work on online treatment, you will have to be able to work on a computer, laptop, smartphone or tablet. The following statements focus on how you have used or are using these types of devices and the internet. Please note that you can also select ‘yes’ if you have done something successfully in the past, but aren’t doing it currently.  I am sending e-mails.   - Yes, I send e-mails - No, I don’t send e-mails   I watch videos online, for example via YouTube or news sites   - Yes, I watch videos online - No, I never watch videos online   I use the internet to read (short) texts such as news articles or blogs.   - Yes, I read texts online - No, I never read texts online   I use social media such as Facebook, Twitter or Instagram.   - Yes, I use social media - No, I don’t use social media   I use the internet to send messages to others, for example via WhatsApp or Facebook Messenger.   - Yes, I send messages via the internet - No, I don’t send messages via the internet | - Perhaps not all questions are as fitting or directly suitable for the use of eMental health interventions. E.g. saving pictures might be less relevant; the focus should be more on skills that are required to use the module (e.g. sending messages, reading short texts, etc.). So the questions should be more on a task-level, and less on a ‘what button to click’-level. - It is important to adapt this question to the characteristics of the main eMental health intervention that is used to make the questions consistent with the required skills. - These questions might not be relevant if patients fill out these questions in the online welcoming module (the fact that they are working on the module indicates that they will possess most of these skills), so these questions might be adapted if they are asked online, possibly by focusing more on how good they are at a task instead of only asking them if they can do it. |
| Part 1b: Possible barriers that might hinder blended treatment (answered by therapist) |  |  |
| 5. Available treatment  Are the treatment objectives clear? And are there treatment modules or parts of the online platform available that match the treatment objectives for this client?  *Clarification*:  Even when there are no modules available, but the therapist is planning to shape blended treatment by means of e-mail, Skype or another generic online method, ‘yes’ can be selected here.   - Yes 🡪 next question - No 🡪 You indicate that the treatment objectives aren’t clear and/or that there are no available parts of the treatment platform that can be used for these objectives. Because of that, no blended treatment can take place. | Not applicable | - According to the project team there always is a fitting module in the eHealth intervention, for example ‘offense chain’, so this question is not very useful. - The treatment objectives should be clear before starting with treatment, so this question is a bit redundant. - According to the project team we should never recommend to not use eMental health since there is always an option, and we don’t want the outcomes of this instrument to be an argument for therapists to not try out eMental health with a patient. |
| 6. Current crisis  Is the client at this point in time in crisis and/or are there severe (concrete) plans for suicide or severe psychotic complaints?  *Clarification*: when is there a crisis? A client requires urgent care (within 24 hours); There is a threatening situation, severe behavioural problems, problems with public order, or supportive factors that are no longer present; There is a forced admission.   - No 🡪 Next question - Yes 🡪 You have indicated that the client is in current crisis. This is a reason to not (yet) start with blended treatment when the complaints are so severe that they prevent contact on a distance. If you see possibilities for contact on a distance, try to make concrete agreements. If this is possible, you can continue with the screening. When care at a distance is not possible, this is a reason to not (yet) start with blended treatment. | Combine with Item 13 on psychosocial problems. | - If there is a current crisis, the patient will probably not be treated in an outpatient setting, but will receive inpatient care. So this item is probably not directly applicable to this specific patient population. - Therapists who participated in the focus group wonder if this really is a contra-indication: if the patient displays severe behavioural problems (which is often the case in forensic psychiatric patients), they might still be able to benefit from these modules.. - This question can be combined with psychosocial problems since they often go hand in hand. By combining these questions, the instrument will also become shorter. |
| 7. Acute medical problems  Are there problems that require immediate medical care, in a way that limit the client to work on his/her treatment? | Not applicable. | - This topic was not mentioned in the interviews and wasn’t considered as a realistic issue by the project team. - If patients require urgent medical care, they probably are also not able to start treatment or use blended care, which makes this question redundant. |
| 8. Intellectual skills (IQ)  Do you think the intelligence level of the client is satisfactory to work with the content of the blended treatment?  *Clarification:* A satisfactory intelligence level for independently working on treatment (especially blended) means that the IQ of a client should be above 80 (above the level of mild intellectual disorder), unless the treatment program is suitable for such target groups. If the IQ is lower than 80, but treatment can be matched to the possibilities of the client, you can click Yes. | **Reading and writing**  In order to work on online treatment, you have to be able to read and write on at least a moderate level. The questions below are about this.  Are you able to read short texts such as articles in newspapers or magazines?   - Yes, I can read well - Yes, but I read with difficulty - No, I can’t read or can hardly read   Are you able to write short texts that are easy to follow for others?   - Yes, I can write clearly - Yes, but I write with difficulty - No, I can’t write or can hardly write | - These questions should be asked to the patient themselves because of the importance of ‘ownership’ and patient-centred care, so this should be moved to Part 1. - The IQ is often very low in this patient population, so by focussing too much on IQ, a large number of patients would be excluded. A focus on the extent to which someone can read and write (which isn’t the same as IQ) is considered to be more valuable according to the interviews and project team, since this is a necessary precondition for working on the eMental health intervention. |
| Part 2a: Possible barriers that might hinder blended treatment (to be discussed by the client and therapist) |  |  |
| Follow-up to Question 4: Internet skills  [If, in internet skills, the patient answers yes less than four times]  You (the client) have indicated not to know how to perform one or more activities that are common in working with the internet. Discuss with your therapist if you (the client) have enough internet skills to work with this program.  *Background information*: Additional matters you can discuss to determine whether the internet skills are satisfactory: (1) Does your client use the computer for work, and how does that go?; (2) Does your client uses e-mail, Facebook or Twitter? How often?; (3) What kind of activities will have to be conducted in treatment (e.g. making assignments, watching videos, sending e-mails, videoconferencing, etc.), would the client be able to do this? | Combined with Question 4 of Part 1. | - This question should be combined with the question on internet skills of Part 1 in order to make the instrument shorter. - This topic should only be discussed if there are severe problems with these skills. This will already become clear from the patient’s answers to the questions of Part 1. |
| 9. Expressing in writing  Discuss: do you (the client) have enough skills to express your feelings in writing for blended treatment? | **2. Writing about thoughts, feelings and behaviour.**  To what extent is a patient able to independently write and reflect on his or her thoughts, feelings and behaviour?  Please note that this is not about the quality of writing, but about the ability to express and describe thoughts and feelings in such a way that it is beneficial for the patient and can be understood by the professional. For each option, it is important that the professional also takes their own consideration about a patient’s abilities into account.   - ***Not or hardly able to express themselves in writing***. The patient indicates not (or hardly) being able to write about thoughts, feelings and behaviour in a way that will benefit their treatment. - ***Moderately able to express themselves in writing***. The patient indicates that they expect to be able to write about thoughts, feelings and behaviour fairly well, or are not entirely sure about their abilities. - ***Able to express themselves in writing***. The patient indicates that they are able to write about thoughts, feelings and behaviour in a way that is beneficial for their treatment. | - This question should be combined with Item 18 on openness in communication from a distance, since these seem to be overlapping constructs. - It is important to also incorporate the perspective from the therapist when selecting the most appropriate option since the project team indicated that patients often overestimate (but sometimes also underestimate) their own skills. - Behaviours and thoughts should be also included in this question since this is also a part of the eMental health intervention, which is (partly) based on cognitive behavioural therapy. - To clarify the difference with Part 1 (on necessary preconditions, amongst which writing skills) it is important to note that this question is not about correct grammar. A patient can be able to express him or herself very well, but the writing might not be perfect. Sometimes patients can even use key terms or short sentences to express themselves. This question focuses more on their ability to reflect independently, without help of a therapist. |
| Part 2b: Possible barriers that might hinder blended treatment (to be discussed by client and therapist) |  |  |
| 10. Motivation and trust  Discuss and answer:  1. Do you (client) trust that a blended treatment can help you with your complaints?  And:  2. Are you (client) motivated to work on a blended treatment? | **1. Motivation for blended treatment**  To what extent is a patient is motivated to work with eMental health in his or her treatment?  Please note that this focuses solely on motivation for online treatment and not for the motivation for the entire (face-to-face) treatment.   - ***Not motivated for online treatment at all.***  The patient indicates that they do not wish to start or continue with online treatment at all. - ***Moderately motivated for online treatment.*** The patient is not entirely sure about starting or continuing but, despite these doubts, might be willing to try. - ***Very motivated for online treatment.*** The patient indicates that they are very willing to start or continue with online treatment and has no to very little doubts. | - Motivation is an important issue in forensic mental healthcare according to the interviews and project team members, so this item should definitely be included. - Lack of trust in effectiveness was not an important topic in the interviews, so this might be removed to shorten the instrument. - It is important to clearly distinguish between treatment motivation in general and motivation for blended care, since a patient might not be motivated for treatment, but might be enthusiastic about the opportunity to work independently on for example an online module. |
| 11. Proneness to crisis  The therapist has indicated that you (the client) are not suicidal or in crisis. Discuss together how likely it is that you (the client) gets in crisis (because of impulsivity, suicidality, emotion regulation problems or the general severity of the complaints). | Not applicable. | - This question should be removed because of the arguments provided in Question 6 on current crisis: it is redundant in this setting. - Discussing the proneness to crisis is not considered to be necessary/particularly relevant for starting blended care by the therapists. |
| 12. Cognitive problems  Do you (the client), besides the problems that were already discussed and for which a treatment plan has already been created, any other complaints that haven’t been discussed yet, such as problems with concentration, planning or forgetfulness?  *Clarification*: These are additional problems, besides the assessment, diagnosis or treatment objectives, that can have a negative effect on blended treatment because the program doesn’t specifically account for this. | **3. Conscientiousness/ working with discipline**  To what extent is a patient capable of sticking to appointments on blended care? This is related to matters such as forgetfulness, concentration, or planning skills.  For each option, it is important that the professional also takes their own consideration about a patient’s conscientiousness into account.   - ***Not disciplined.*** The patient indicates having much trouble with sticking to agreements, either consciously or unconsciously, and has a lot of difficulty with working on assignments individually. - ***Moderately disciplined***. The patient indicates to fluctuate in the extent to which they stick to agreements, and sometimes has difficulty with working on assignments individually. - ***Disciplined***. The patient indicates that they (almost) always stick to agreements and is capable to work on assignments individually. | - Based on the interviews, the focus of this item should be shifted: not directly related to cognitive problems such as problems with attention or concentration, but to the consequences of these types of problems. The focus now lies on starting on and finishing assignments in the intended way (which might indeed be influenced by cognitive problems related to for example ADHD, but this does not necessarily have to be the case). This makes this item a bit more concrete and broadly applicable. - According to the project team, it is important to discuss this with the patient, but also to incorporate the estimation of the therapist and regularly re-evaluate since the patient’s estimation of their own conscientiousness might not always be accurate. |
| 13. Psychosocial problems  Discuss if you (the client) have psychosocial problems that can negatively affect or disturb the blended treatment.  *Clarification:* These are for example relationship problems, problems within the family, fighting with the neighbours, financial problems, etc. | **4. Psychosocial problems**  To what extent are there problems in the patient’s personal life and/or severe psychiatric disorders that can have a negative impact on using the eMental health intervention? Examples are relationship or family problems, severe financial problems, current psychosis, et cetera.  For each option, it is important that the professional also takes their own consideration about a patient’s psychosocial problems and their impact on blended care into account.   - ***Many and/or severe problems***. The patient indicates that there are psychiatric or personal issues that are expected to have a severe negative impact on online treatment and are expected to prevent the patient from working on online treatment individually. - ***Several and/or moderately severe problems***. The patient indicates that there are psychiatric or personal issues that are expected to have a moderately negative influence on online treatment and are expected to partially prevent the patient from working online treatment individually. - ***No or almost no problems***. The patient indicates that there are no or very few psychiatric or personal issues, and the existing issues are not expected to negatively impact online treatment. | - In this item, the psychological problems (current crisis) that are referred to in the omitted items 6 and 11 are integrated in order to prevent overlap and to shorten the instrument. The focus on psychological problems is added to the explanation of this item. - This item focuses on all problems (social or psychological) that might hinder treatment. Because this is quite broad, examples are added, which are derived from the interviews. |
| 14. Additional barriers  Discuss if there are additional characteristics/things that can disturb the communication and blended treatment.  *Clarification:* Think of co-morbidity that hasn’t been discussed yet in earlier parts. | Not applicable | - This item was removed to make the instrument as short as possible. It is expected that additional barriers, if applicable, will arise during the conversation based on the new items. |
| Part 3: Possible facilitators that can facilitate blended treatment. (to be discussed by client and therapist) |  |  |
| 15. Working alliance  Is there a good working alliance, or do you (therapist and client) expect that a good working alliance will be developed?  Note: Here it is important that you (the client) recognize your own contribution to the therapy and are aware of what is expected of you. | Not applicable | - The person administering this instrument might be someone else than therapist (e.g. the ‘intaker’), so this question does not always fit the situation. - This is hard to assess in the first meeting(s), especially in forensic psychiatric patients who are obliged to attend treatment; they might have a different attitude than later in the treatment process. |
| 16. Preference because of practical reasons  Do you (the client) have a preference for blended treatment because of practical reasons?  *Clarification:* Think, for example, about a busy job, long traveling time, less traveling costs. | Not applicable | - The topics of this question will also be discussed in the new question on motivation, which makes this question a bit redundant. - Because of the need to shorten the instrument, this question can be removed. |
| 17. Preference because of fear, safety, stigma  Do you (the client) have a preference for blended care because of fear, safety or stigma?  *Clarification:* Not all clients want others to know that they receive mental healthcare because of stigmatization. It can be difficult for them to go to a mental healthcare building or to ask for time off for treatment. The fear to go outside or to travel with public transport might also be a barrier for regular, face-to-face treatment. | Not applicable | - Because of the need to shorten the instrument, this question can be removed. - The answers to this question are expected to overlap with the topics that arise when discussing the item on motivation. |
| 18. Openness in communication from a distance  Do you (the client) think that you will be able to communicate as open as or even more open in online contact compared to in-person contact?  *Clarification:* The following activities can indicate that you (the client) are able to communicate open in online contact: (a) did you ever keep a diary?; (b); do you like to write?; (c) are you shy in personal contact; (d) do you like using social media (Facebook, Twitter, chatrooms, online fora) to exchange emotions and thoughts with others? | Integrated with Item 2 on Writing about thoughts, feelings and behaviour | - The topics that will be discussed in this question are expected to overlap with the new item on Writing about thoughts, feelings and behaviour, since this item focuses on the ability of a patient to independently think about these types of topics and to write them down in order for the therapist to read them. |
| 19. Discipline  Are you (the client) disciplined and conscientious?  *Clarification:* For example, do you stick to agreements? | Combined with Item 3 on Conscientiousness/working with discipline | - This item is combined with the (old) item on cognitive problems in order to make a more comprehensive item, since it is expected that the cognitive problems contribute to a lack of discipline in the patient, based on the interviews. |
| 20. Social support  Are there persons in your (the client) environment (partner, family member, friends, neighbours) that can support and encourage you in the blended treatment? | **5. Social support**  To what extent does the patient have a social support system, e.g. partner, parents or friends, that are willing and able to support them in online treatment?   - ***No social support.*** The patient indicates that they have no people within their social environment that are willing and able to support them in online treatment, or that they do not have access to a social support system. - ***Moderate social support.*** The patient indicates that they have some people within their social environment that might be willing and able to support them in online treatment. - ***Much social support***. The patient indicates that many people within their social environment that are willing and able to support them in online treatment. | - According to the participants of the interviews, this is an important item, so it should be maintained. - The project team indicated that there are multiple ways in which loved ones can be involved in the online modules. This item can help therapist and patient in discussing how their loved ones can be actively involved in their (online) treatment, since this often does not happen. |
