# Supplementary material for: Shaping Blended Care: Adapting an Instrument to Support Therapists in Using eMental Health
Source: JMIR Ment Health. 2020 Nov 13;7(11):e24245. doi: 10.2196/24245 (PMC7695535; doi:10.2196/24245)
Supplement: Multimedia Appendix 2 [file mental_v7i11e24245_app2.docx]

# Multimedia Appendix 2

## Fit for Blended Care instrument – Therapist-centered version

This instrument consists of two parts: one to be filled out by the patient before treatment, and one that should be filled out during treatment, as part of a conversation on shaping blended care. This instrument is specifically tailored to forensic mental healthcare and cannot simply be copy-pasted to other types of healthcare. Furthermore, if this instrument is used in other organizations, terms can be adapted to fit the jargon used in different organizations (e.g. eHealth instead of online treatment, or the name of the specific intervention that is used).

**Part 1: To be filled out by the patient**

At [this organization], there are possibilities to work individually on your treatment via a website and mobile apps, as an addition to your face-to-face appointments. With these questions we want to determine if we can offer these opportunities from a practical point of view. Your therapist will discuss this with you later in the process in more detail. Please indicate for each question which option fits you best.

**Reading and writing**

In order to work on online treatment, you have to be able to read and write on at least a moderate level. The questions below are about this.

Are you able to read short texts such as articles in newspapers or magazines?

- Yes, I can read well
- Yes, but I read with difficulty
- No, I can’t read or can hardly read

Are you able to write short texts that are easy to follow for others?

- Yes, I can write clearly
- Yes, but I write with difficulty
- No, I can’t write or can hardly write

**Place to work on online treatment**

These questions focus on whether you have a suitable place to work on your treatment on a computer, laptop, smartphone or tablet.

Do you have access to a device (computer, laptop, smartphone or tablet) on which you are able and willing to work on online treatment?

- Yes, I do have access to a suitable device
- No, I don’t have access to a suitable device
- No, but I will have access to a suitable device within approximately one month

Do you have access to a good, stable internet connection?

- Yes, I have access to good internet
- No, I don’t have access to good internet
- No, but I will have access to good internet within approximately one month

Do you have access to a place where you can work in a calm and pleasant way on your online treatment?

- Yes, I do have access to a suitable place
- No, I don’t have access to a suitable place
- No, but I will have access to a suitable place within approximately a month

**Technology usage**

In order to work on online treatment, you will have to be able to work on a computer, laptop, smartphone or tablet. The following statements focus on how you have used or are using these types of devices and the internet. Please note that you can also select ‘yes’ if you have done something successfully in the past, but aren’t doing it currently.

I am sending e-mails.

- Yes, I send e-mails
- No, I don’t send e-mails

I watch videos online, for example via YouTube or news sites

- Yes, I watch videos online
- No, I never watch videos online

I use the internet to read (short) texts such as news articles or blogs.

- Yes, I read texts online
- No, I never read texts online

I use social media such as Facebook, Twitter or Instagram.

- Yes, I use social media
- No, I don’t use social media

I use the internet to send messages to others, for example via WhatsApp or Facebook Messenger.

- Yes, I send messages via the internet
- No, I don’t send messages via the internet

Thank you very much for answering these questions! Your therapist will further discuss the possibilities of online treatment with you during treatment.

**Part 2: To be filled out by the therapist**

Welcome to the second part of the Fit for Blended care instrument for mental healthcare for forensic psychiatric outpatients. This instrument offers tools to discuss and determine how to shape blended treatment together.

At this point, the patient is expected to have filled out the first part of this instrument, which focuses on necessary preconditions for online treatment. In this second part, five factors are provided that can have a positive or negative impact on online treatment. These five factors are based on research from the University of Twente and are formulated in close cooperation with experienced therapists.

You will discuss these five factors with the patient, and based on the outcomes of these conversations, you can indicate to what extent a factor is present within a patient by choosing one of the three answering options. It is important to consider both the patient’s and your own assessment and come to a balanced decision.

You can use this instrument at the start of and during treatment. You can use it multiple times, based on your own estimation of its added value for treatment at that specific point in time. The main goal of this instrument is to start a conversation on shaping blended care in order to facilitate shared-decision making; its goal is not to give concrete advise about whether and how to shape blended treatment, that is up to you and the patient.

**1. Motivation for blended treatment**

To what extent is a patient is motivated to work with eMental health in his or her treatment?

Please note that this focuses solely on motivation for online treatment and not for the motivation for the entire (face-to-face) treatment.

- ***Not motivated for online treatment at all.*** The patient indicates that they do not wish to start or continue with online treatment at all.
- ***Moderately motivated for online treatment*.** The patient is not entirely sure about starting or continuing but, despite these doubts, might be willing to try.
- ***Very motivated for online treatment*.** The patient indicates that they are very willing to start or continue with online treatment and has no to very little doubts.

**2. Writing about thoughts, feelings and behaviour.**

To what extent is a patient able to independently write and reflect on his or her thoughts, feelings and behaviour?

Please note that this is not about the quality of writing, but about the ability to express and describe thoughts and feelings in such a way that it is beneficial for the patient and can be understood by the professional. For each option, it is important that the professional also takes their own consideration about a patient’s abilities into account.

- ***Not or hardly not able to express themselves in writing***. The patient indicates not (or hardly not) being able to write about thoughts, feelings and behaviour in a way that will benefit their treatment.
- ***Moderately able to express themselves in writing***. The patient indicates that they expect to be able to write about thoughts, feelings and behaviour fairly well, or are not entirely sure about their abilities.
- ***Able to express themselves in writing***. The patient indicates that they are able to write about thoughts, feelings and behaviour in a way that is beneficial for their treatment.

**3. Conscientiousness/working with discipline**

To what extent is a patient capable of sticking to appointments on blended care? This is related to matters such as forgetfulness, concentration, or planning skills.

For each option, it is important that the professional also takes their own consideration about a patient’s conscientiousness into account.

- ***Not disciplined.*** The patient indicates having much trouble with sticking to agreements, either consciously or unconsciously, and has a lot of difficulty with working on assignments individually.
- ***Moderately disciplined***. The patient indicates to fluctuate in the extent to which they stick to agreements, and sometimes has difficulty with working on assignments individually.
- ***Disciplined***. The patient indicates that they (almost) always stick to agreements and is capable to work on assignments individually.

**4. Psychosocial problems**

To what extent are there problems in the patient’s personal life and/or severe psychiatric disorders that can have a negative impact on using the eMental health intervention? Examples are relationship or family problems, severe financial problems, current psychosis, et cetera.

For each option, it is important that the professional also takes their own consideration about a patient’s psychosocial problems and their impact on blended care into account.

- ***Many and/or severe problems***. The patient indicates that there are psychiatric or personal issues that are expected to have a severe negative impact on online treatment and are expected to prevent the patient from working on online treatment individually.
- ***Several and/or moderately severe problems***. The patient indicates that there are psychiatric or personal issues that are expected to have a moderately negative influence on online treatment and are expected to partially prevent the patient from working online treatment individually.
- ***No or almost no problems***. The patient indicates that there are no or very few psychiatric or personal issues, and the existing issues are not expected to negatively impact online treatment.

**5. Social support**

To what extent does the patient have a social support system, e.g. partner, parents or friends, that are willing and able to support them in online treatment?

- ***No social support.*** The patient indicates that they have no people within their social environment that are willing and able to support them in online treatment, or that they do not have access to a social support system.
- ***Moderate social support.*** The patient indicates that they have some people within their social environment that might be willing and able to support them in online treatment.
- ***Much social support***. The patient indicates that many people within their social environment that are willing and able to support them in online treatment.
