# Supplementary material for: Shaping Blended Care: Adapting an Instrument to Support Therapists in Using eMental Health
Source: JMIR Ment Health. 2020 Nov 13;7(11):e24245. doi: 10.2196/24245 (PMC7695535; doi:10.2196/24245)

# Multimedia Appendix 3

## Fit for Blended Care instrument - Patient-centered version

The following five items are similar to the five in the therapist-centred version in terms of content, but are formulated with the patient as main target group. They are meant as a discussion tool to facilitate shared-decision making for therapist and patient. This means that the patient does not answer these questions individually; options are selected based on a conversation with therapist.

There are multiple ways to present these questions to the patient: a printed out version of the questionnaire which can be filled out on paper (see Figure 1) and later entered into the electronic patient file an online version which can be filled out directly in the electronic patient file with patient and therapist both sitting behind the computer, and five discussion cards which can serve as input for a conversation, where the answers again have to entered in the electronic patient file at a later point in time. Please select the way to discuss these questions that fits your patient best. If necessary, you can give additional explanation for the factors, using the therapist-centred version of the instrument.

**1. Motivation**

I want to work with online interventions in my treatment.

- Yes, I want that.
- It doesn’t matter.
- No, I don’t want that.

**2. Reflecting on thoughts, feelings and behaviour**

I can write about what I think, feel and do. (Please note that this does not refer to flawless grammar, but to the extent that writing about this can be beneficial for you.)

- Yes, I can do that.
- I can do that to some extent.
- No, I cannot do that.

**3. Sticking to agreements**

I will always complete individual assignments in online treatment in the way that I agreed upon with my therapist.

- Yes, I will do that.
- I sometimes will, and sometimes won’t do that.
- No, I will not or hardly do that.

**4. Influence of personal circumstances**

At this point in my life, there are multiple things going on that will prevent me from working on online treatment.

- No, there aren’t any problems that will influence how I will work on online treatment.
- There are some problems that might have some negative impact, but will not prevent me completely from working online treatment.
- Yes, there are problems that will definitely influence how I will work on online treatment.

**5. Social support**

People from my social environment can help me with working on my online treatment.

- Yes, there are many people that can support me in working on online treatment.
- There might be several people that can support me in working on online treatment.
- No, there are no people that can support me in working on online treatment.

*Figure 1.* A screenshot of a possible design of the patient-centred version of the Fit for Blended Care instrument.


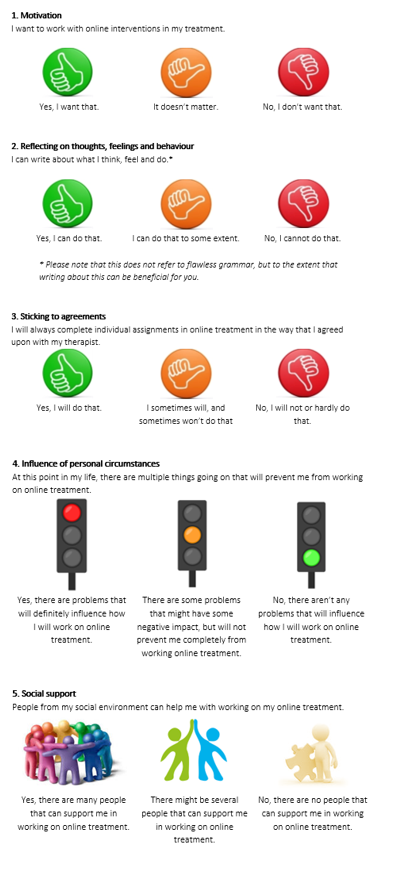

Supplement: Multimedia Appendix 3 [file mental_v7i11e24245_app3.docx]
